# Analysis of the Spinopelvic Parameters in Patients with Fragility Fractures of the Pelvis

**DOI:** 10.3390/jcm12134445

**Published:** 2023-07-02

**Authors:** Moritz F. Lodde, Thies M. Fischer, Oliver Riesenbeck, Steffen Roßlenbroich, J. Christoph Katthagen, Daria Rometsch, Dennis Günes, Marc Schneider, Michael J. Raschke, Ulf Liljenqvist

**Affiliations:** 1 Department for Trauma Hand and Reconstructive Surgery, University Hospital Münster, Albert-Schweitzer-Campus 1, Building W1, Waldeyerstraße 1, 48149 Münster, Germany; oliver.riesenbeck@ukmuenster.de (O.R.); steffen.rosslenbroich@ukmuenster.de (S.R.); christoph.katthagen@ukmuenster.de (J.C.K.); michael.raschke@ukmuenster.de (M.J.R.); 2 Department for Spine Surgery and Scoliosis, St. Franziskus-Hospital GmbH Münster, Hohenzollernring 70, 48145 Münster, Germany; thiesmartin.fischer@sfh-muenster.de (T.M.F.); dennis.guenes@sfh-muenster.de (D.G.); marc.schneider@sfh-muenster.de (M.S.); ulf.liljenqvist@sfh-muenster.de (U.L.); 3 Department of Otorhinolaryngology, Head and Neck Surgery, University Hospital Münster, Kardinal-von-Galen-Ring 10, 48149 Münster, Germany; daria.rometsch@ukmuenster.de

**Keywords:** FFPs (fragility fractures of the pelvis), osteoporosis, spinopelvic parameters, pelvic incidence, pelvic tilt, sacral slope, lumbar lordosis index, sagittal balance

## Abstract

Background: We investigated the spinopelvic parameters of lumbar lordosis (LL), pelvic incidence (PI), pelvic tilt (PT) and sacral slope (SS) in patients with fragility fractures of the pelvis (FFPs). We hypothesized that these parameters differ from asymptomatic patients. Methods: All patients treated for FFPs in a center of maximal care of the German Spine Society (DWG) between 2017 and 2021 were included. The inclusion criteria were age ≥ 60 years and the availability of a standing lateral radiograph of the spine and pelvis in which the spine from T12 to S1 and both femoral heads were visible. The baseline characteristics and study parameters were calculated and compared with databases of asymptomatic patients. The two-sample *t*-Test was performed with *p* < 0.05. Results: The study population (*n* = 57) consisted of 49 female patients. The mean age was 81.0 years. The mean LL was 47.9°, the mean PT was 29.4°, the mean SS was 34.2° and the mean PI was 64.4°. The mean value of LLI was 0.7. LL, LLI and SS were significantly reduced, and PI and PT were significantly increased compared to asymptomatic patients. Conclusions: The spinopelvic parameters in patients with FFPs differ significantly from asymptomatic patients. In patients with FFPs, LL, LLI and SS are significantly reduced, and PI and PT are significantly increased. The sagittal spinopelvic balance is abnormal in patients with FFPs.

## 1. Introduction

**Figure 1:**
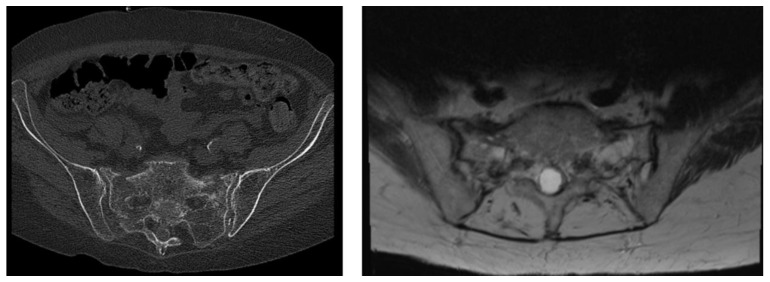
An 82-year-old patient presenting with a fragility fractures of the pelvis (FFP) type IV or OF4 (OF-Pelvis classification) with displaced bilateral posterior lesions. The bilateral fractures are seen in the computer tomography (CT) (**left side**) and magnetic resonance imaging (MRI) (**right side**). The patient suffered from a low-energy trauma, falling on her back.

The incidence, awareness and surgical treatment of fragility fractures of the pelvis (FFPs) has increased substantially during the last few decades [1,2,3]. This entity of insufficiency fractures was first described by Lourie in 1982 when reporting on three cases of spontaneous fractures of the sacrum in patients with osteoporosis [4]. FFPs are associated with osteoporosis [5]. FFPs are caused by low-energy trauma such as a ground-level fall and can be observed in complete absence of a trauma [5,6,7]. The importance of this type of insufficiency fracture is highlighted by the demographic development in industrialized countries with a projected significant increase in people older than 65 years [8]. The FFP classification system published by Rommens et al. [9] and the OF-Pelvis classification of osteoporotic sacral and pelvic ring fractures [10] describe the different fracture types (Figure 1).

**Figure 2:**
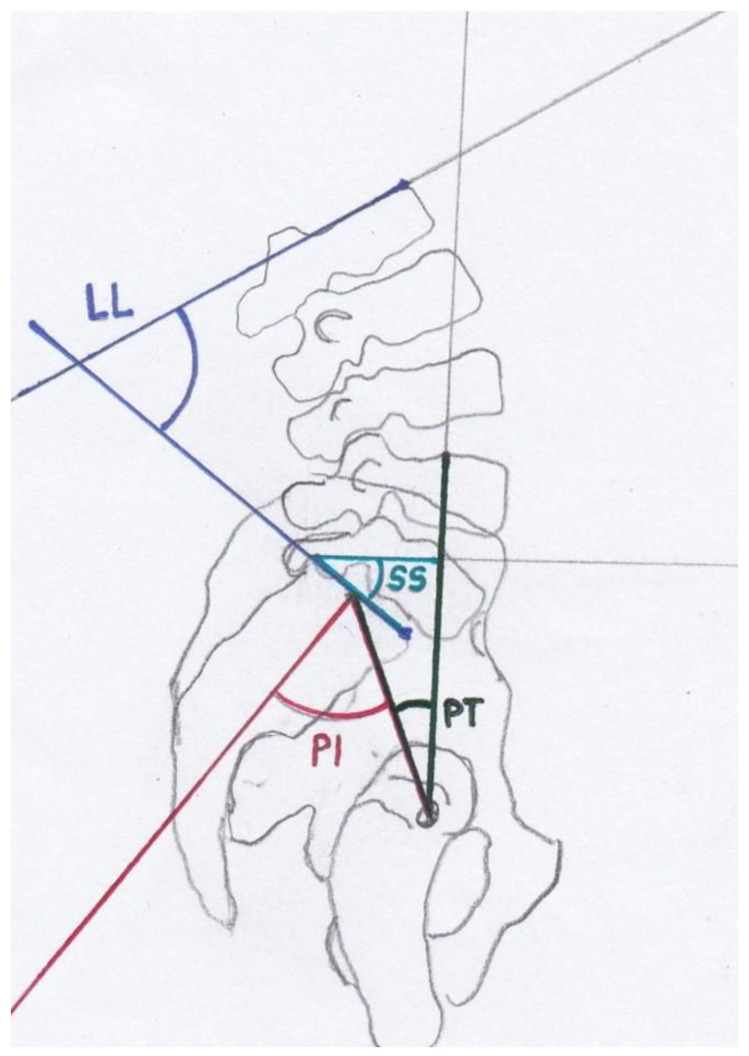
Modified after [23,24]: The spinopelvic parameters are lumbar lordosis (LL), pelvic incidence (PI), pelvic tilt (PT) and sacral slope (SS).

The restoration of mobility and pain reduction are the most important aims [11,12]. Several surgical treatment options exist and have been biomechanically tested [1,12,13,14,15,16,17]. Surgical treatment is recommended for patients suffering from type III and IV FFPs, while optimal treatment strategies for patients suffering from type II FFPs are discussed [1]. For patients with FFP II, surgical intervention is recommended after failing a brief period of non-surgical treatment [18]. Vertical sacral ala fractures, fracture dislocations of the sacroiliac joint and spinopelvic dissociations should be treated with operative stabilization [5]. FFPs present an injury of the lumbosacral junction. The lumbosacral junction [19] and the spinopelvic parameters [20] have been subjects of research for almost 100 years. The disturbed condition of these parameters resulting in spondyloptosis (corresponding to Meyerding grade IV spondylolisthesis) was first described in 1782 by the Belgian obstetrician Herbinaux [21]. Spinopelvic parameters determining the sagittal balance are lumbar lordosis (LL), pelvic incidence (PI), pelvic tilt (PT), sacral slope (SS) and the lumbar lordosis index (LLI,(ratio lumbar lordosis/pelvic incidence)) (Figure 2) [22].

PI was described by Duval-Baupère et al. [25,26] and determines pelvic orientation and the size of LL. PI is the angle between a line joining the axis of the femoral heads to the midpoint of the sacral plate and a perpendicular line to the sacral plate (Figure 2). PI determines the spatial position of the pelvis in the standing position [23] and remains constant in adult persons [25,26,27]. PI differs significantly in patients with spondylolisthesis from the asymptomatic population, and an increase in PI correlates with the Meyerding grading of spondylolisthesis [23,28]. A high PI leads to a concentration of stress at the L5-S1 junction [29]. The sum of PT and SS is PI [25,30]. PT and SS are position-dependent variables which determine the orientation of the pelvis in the sagittal plane [23]. Consequently, the sum of PT and SS correlates with PI. A low PT indicates an anteverted pelvis, and a high PT indicates a retroverted pelvis [31]. LLI is the ratio between LL and PI [22]. LLI correlates with the lack of lordosis and is a valuable additional radiographic parameter [22]. LLI matches LL with PI [22]. PI is specific for each individual and correlates with LL, but it is not affected by aging [22]. In adult patients with scoliosis, an LLI < 0.5 was always associated with a vertebral osteotomy, whereas patients with an LLI > 0.5 were treated without osteotomy [22].

To the best of our knowledge, there are no studies analyzing the interaction between the pelvis and the sagittal alignment of the spine in patients with FFPs. The aim of this study was to investigate the spinopelvic parameters LL, PI, PT and SS, in patients with FFPs. We hypothesized that the spinopelvic parameters differ in patients with FFPs, leading to an abnormal sagittal balance.

## 2. Materials and Methods

**Figure 3:**
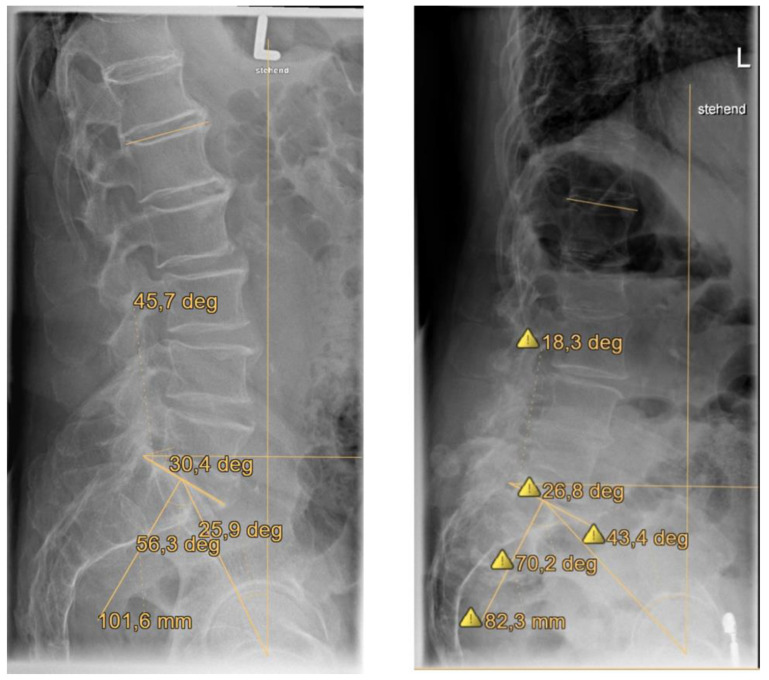
Spinopelvic parameters of LL, PT, SS and PI were analyzed for all patients. Measurement was conducted in a standing lateral radiograph of the spine and pelvis in which the spine from T12 to S1 and both femoral heads were visible.

All patients treated for FFPs in a center of maximal care of the German Spine Society (DWG) between 2017 and 2021 were included. The inclusion criteria of this retrospective study were a diagnosis of an FFP according to the classification of Rommens et al. [9], an age equal to or over 60 years and the availability of a standing lateral radiograph of the spine and pelvis in which the spine from T12 to S1 and both femoral heads were visible. The availability of computer tomography (CT) or magnetic resonance imaging (MRI) of the pelvis (Figure 1) was a further inclusion criterion. The exclusion criteria were the diagnosis of osteoporotic vertebral fractures, degenerative diseases of the spine like spondylolisthesis or spondyloptosis, patients with significant lower limb abnormalities, patients with any previous spine surgery and patients with any associated musculoskeletal syndrome [23,31]. Out of 507 initially identified patients treated for lumbar spine or pelvic ring pathologies, a total of 57 patients were included according to the inclusion criteria. After classifying all CT or MRI of the pelvises according to Rommens et al. [9] and according to the OF-Pelvis classification system [10], all digitalized radiographs were analyzed by two investigators independently (M.F.L. MD, resident 5th year; T.M.F. MD, consultant) (Appendix A Table A1) [23,31]. Type I FFPs are anterior lesions only, type II FFPs are non-displaced posterior lesions, type III FFPs are displaced unilateral posterior lesions and type IV FFPs are displaced bilateral posterior lesions. The OF-Pelvis classification of osteoporotic sacral and pelvic ring fractures distinguishes five subgroups and underlines the importance of magnetic resonance imaging (MRI) for the diagnosis of as FFP [10]. OF1 is a pelvic ring edema with no fracture visible in computer tomography (CT). OF2 defines anterior pelvic ring fractures, OF3 identifies unilateral sacral fractures, OF4 is bilateral sacral fractures and OF5 is iliac or sacroiliac fractures (Figure 1) [10]. Using the software programs Picture Archiving and Communications System (PACS) and digital picture analysis JiveX DICOM viewer (VISUS Health IT GmbH, Bochum, Germany), the fast and exact calculation of the spinopelvic parameters LL, PT, SS and PI was conducted via the interactive measurement of the anatomic landmarks of the spine, pelvis and femoral heads (Figure 3).

Additionally, LLI was calculated. Using a statistical software program (IBM SPSS Statistics v.23, IBM, Armonk, NY, USA), the baseline characteristics of the patients and mean values and standard deviations for LL, PI, PT, SS and LLI were calculated. The mean values and standard deviations (SDs) for LL, PI, PT, SS and LLI were compared with published databases of asymptomatic patients of Schwab [32,33], LeHuec [34] and Barrey-Roussouly [35]. The mean values were calculated for better comparison with the previously published studies, which reported mean values. The two-sample *t*-Test was performed with *p* < 0.05. This study was approved by the local ethics committee (the ethics committee of the Medical Association of Westfalen-Lippe, no: 2022-573-f-S). Due to the retrospective study design, informed consent was not required.

## 3. Results

### 3.1. Baseline Characteristics

The study population (*n* = 57) consisted of 49 female patients (86.0%) and 8 male patients (14.0%). The mean age was 81.0 years (interquartile range (IQR) 77.1–84.8). The mean age of the female patients (*n* = 49) was 80.9 years (IQR 76.9–85.3), and it was 81.5 years (IQR 79.1–84.9) for the male patients (*n* = 8) (Table 1).

### 3.2. Distribution of FFP Types According to the FFP Classification System [9] and OF-Pelvis Classification System [10]

**Figure 4:**
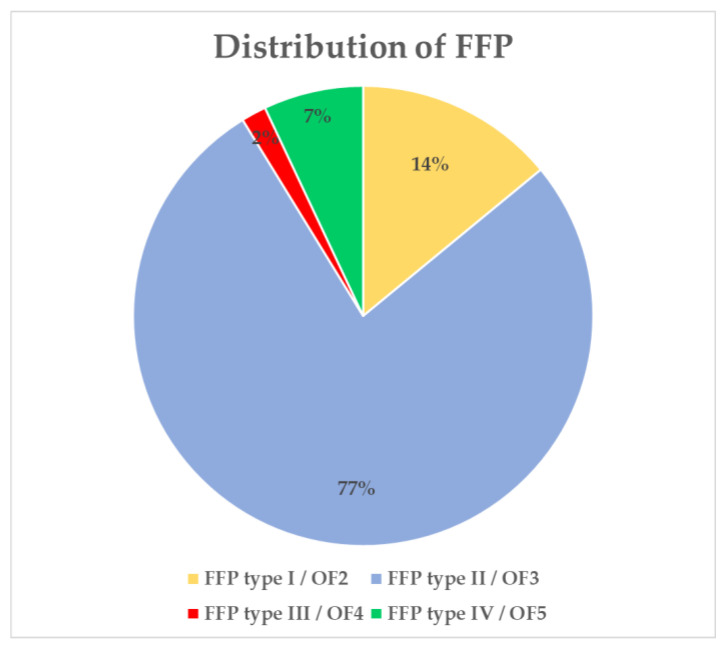
Type II FFP (fragility fracture of the pelvis) was observed in 44 patients (77.2%) according to the FFP classification system [9] and OF-Pelvis classification system [10].

Type II FFP or OF3 occurred in 44 patients (77.2%) (Figure 4). Type I FFP or OF2 were observed in eight patients (14.0%), type III FFP or OF4 in one patient (1.8%) and type IV FFP or OF5 in four patients (7.0%) (Figure 4).

### 3.3. Spinopelvic Parameters

The study population (*n* = 57) had a mean LL of 47.9° (standard deviation SD ± 14.4), a mean PT of 29.4° (SD ± 8.8°), a mean SS of 34.2° (SD ± 10.4°) and a mean PI of 64.4° (SD ± 12.9°) (Table 2).

**Figure 5:**
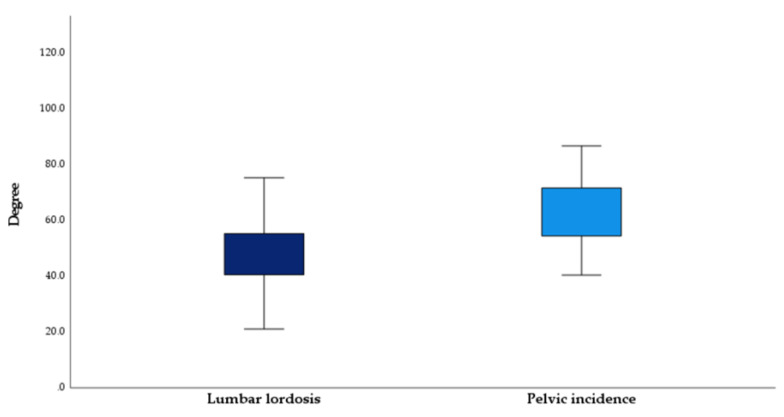
The mean lumbar lordosis and pelvic incidence of the study’s population were significantly (*p* < 0.01) different.

The mean value of LLI was 0.7 (SD ± 0.2). The difference between PI and LL was 16.5° and thus statistically significant (*p* < 0.01) (Figure 5).

No statistically relevant correlations were detected between the FFP subtypes I–IV and LL (*p* = 0.93), PI (*p* = 0.47), SS (*p* = 0.83) or PT (*p* = 0.94).

### 3.4. Spinopelvic Parameters in the Female and Male Subpopulation

The female patients (*n* = 49) had a mean LL of 48.0° (SD ± 14.6°), a mean PT of 29.1° (SD ± 8.3°), a mean SS of 34.4° (SD ± 10.8°) and a mean PI of 64.2° (SD ± 12.3°). The female patients had a mean LLI of 0.8° (SD ± 0.2°). The male patients (*n* = 8) had a mean LL of 47.1° (SD ± 14.0°), a mean PT of 31.3° (SD ± 11.9°), a mean SS of 32.6° (SD ± 7.9°) and a mean PI of 65.9° (SD ± 17.2°). The male patients had a mean LLI of 0.7 (SD ± 0.1). No statistically significant differences in the examined parameters were detected regarding the female and male patients (*p* > 0.05).

### 3.5. Spinopelvic Parameters of FFP Patients Compared to Asymptomatic Patients

**Figure 6:**
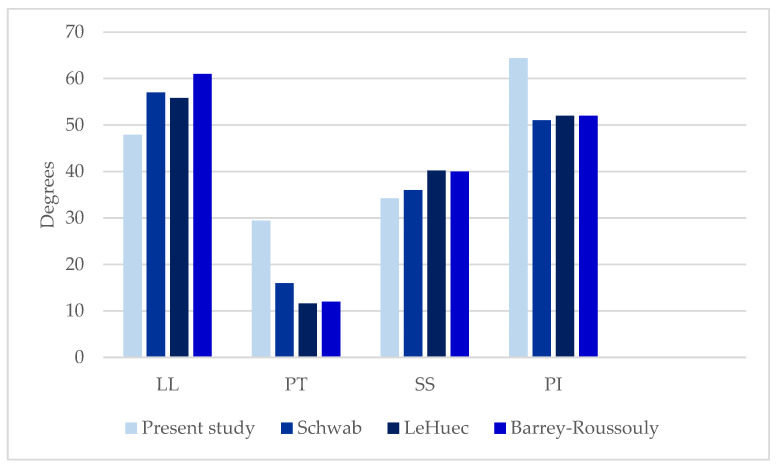
The spinopelvic parameters in the present study compared to the spinopelvic parameters in previous studies. LL (47.9°) and SS (34.2°) in the present study were significantly reduced (*p* < 0.01). PI (64.4°) and PT (29.4°) were significantly increased (*p* < 0.01).

**Figure 7:**
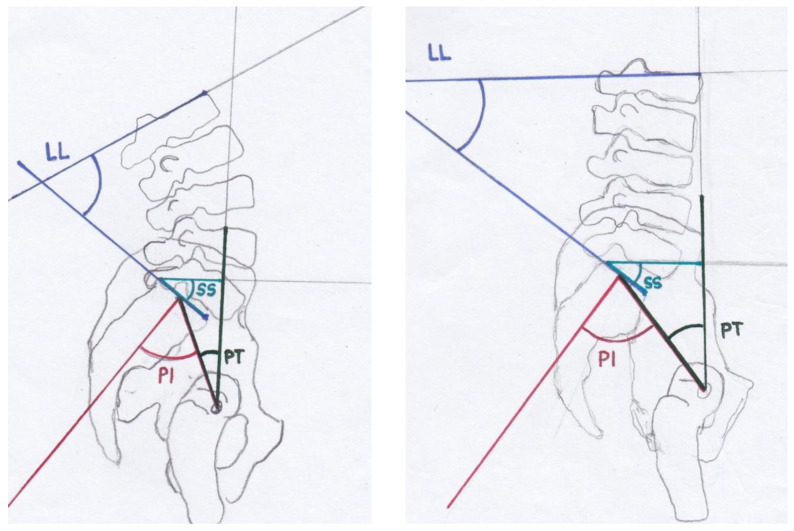
Modified after [23,24]: The spinopelvic parameters of asymptomatic patients are drawn on the left side. The spinopelvic parameters of patients suffering from FFPs are drawn on the right side. In patients with FFPs, LL and SS are reduced, whereas PI and PT are increased. A higher PT and lower SS result from the backward rotation of the pelvis as a compensatory mechanism.

The LL of the present study (47.9° SD ± 14.4°) was significantly lower compared to the LL of asymptomatic patients published in studies of Schwab [33], LeHuec [34] and Barrey-Roussouly [35] (*p* < 0.01) (Table 3; Figure 6 and Figure 7).

The PT (29.4° SD ±8.8°) was significantly higher than the PT of asymptomatic patients in previously published studies [33,34,35] (*p* < 0.01). The SS (34.2° SD ± 10.4°) was significantly lower compared to the results of LeHuec [34] and Barrey-Roussouly [35] (*p* < 0.01). There was no significant difference between the SS of the present study and the SS of Schwab [33]. The PI (64.4° SD ± 12.9°) was significantly higher in the population of the present study (*p* < 0.01) [33,34,35].

## 4. Discussion

The present study examined the spinopelvic parameters in 57 patients with FFP. To the best of our knowledge, this study describes the spinopelvic parameters of the largest cohort of FFP patients to date. The main results are that LL and LLI are substantially reduced in patients with FFPs compared to asymptomatic patients. PT and PI are significantly increased in patients with FFPs. The baseline characteristics of the study population are typical for patients with FFPs. A mean age of 81.0 years and the gender distribution—86% female patients—of the present study are in accordance with the results of previously published studies [1,6,11,36,37]. Additionally, the distribution of FFP types, or classifications according to the OF-pelvis classification system, in this study is comparable to the data of prior studies [18,36,37].

PI defines the anatomic composition of the pelvis and the sagittal shape of the spine (Figure 2, Figure 3, Figure 6 and Figure 7) [25,26]. This spinopelvic parameter describes the spinopelvic balance. The PI of the present study was 64.4° (SD ± 12.9°) and significantly higher (*p* < 0.05) compared to the PI of asymptomatic patients (Figure 7) [33,34,35]. The sagittal shape of the spine correlates with the incidence of lower back pain [38]. PI correlates with the incidence of spondylarthrosis [25], and an increased PI was detected in patients with spondyloptosis [39]. In addition, PI is significantly higher in patients with low- and high-grade spondylolisthesis [23,28]. A high PI effects a concentration of biomechanical stress at the L5-S1 junction [29] and is associated with osteoporosis [40]. An FFP occurs after low-energy trauma or in the absence of any trauma [4] and is also associated with osteoporosis [5,6,7]. As an increased PI correlates with spondylarthrosis, spondylolisthesis and increased biomechanical stress at the L5-S1 junction, it might lead to the development of fractures of the sacrum after low-energy trauma or even spontaneously in the presence of reduced bone quality. More studies evaluating this hypothesis and possible correlations are needed. In patients with FFPs, the PI is significantly increased. An increased PI might be a risk factor for FFPs. The LL was 47.9° (SD ± 14.4°) and significantly lower compared to the LL of asymptomatic patients (Figure 6 and Figure 7) [33,34,35]. In asymptomatic patients, PI and LL correlate strongly [26,27]. PI is essential to determine the appropriate LL in a patient [25,26,30,41,42,43,44]. Schwab et al. [32] established the formula of LL = PI ± 9°, which was verified by several studies [45,46,47,48,49,50]. The risk for adjacent segment disease, proximal junctional kyphosis and lumbopelvic fixation failure [49,51,52,53] is increased in cases of a mismatch of LL = PI ± 9°. In addition, sacral fractures after lumbosacral fusion are observed more frequently in patients with a mismatch [54]. Interestingly, in our cohort, LL values did not match the formula of Schwab et al. [32]. Thus, a mismatch of the formula LL = PI ± 9° exists in patients with FFPs, indicating a further risk factor for FFPs. The LLI of the present study (0.7 ± 0.2) was substantially smaller than the LLI of previously published studies [33,34,35]. LLI matches LL with PI [22], and it is a further validated radiographic parameter evaluating spinal alignment [22]. In the present study, the PT (29.4° ± 8.8°) was significantly (*p* < 0.05) higher than that in asymptomatic patients (Figure 6 and Figure 7) [33,34,35]. The SS (34.2 ± 10.4) was significantly (*p* < 0.05) lower [34,35]. An analysis of spinopelvic alignment in patients with lumbar disc herniation showed a lower LL and SS and a higher PT due to a loss of lumbar lordosis and sagittal imbalance [55]. In patients with osteoporotic vertebral fractures, a PT ≥ 27° is associated with surgical intervention due to the failure of conservative treatment [56]. PT and the local kyphotic angles have significant influence on the treatment of osteoporotic vertebral fractures [56]. In elderly patients, lumbar lordosis is often reduced and thoracic kyphosis is increased [57,58]. The decompensation of the spine results in back pain and vertebral fractures [59,60]. A higher PT and lower SS result from the backward rotation of the pelvis as a compensatory mechanism (Figure 7). In patients with FFPs, PT and SS are significantly different from asymptomatic patients as a compensatory mechanism of an existing sagittal imbalance. An increased PT and a reduced SS might be risk factors for FFPs.

The present study evaluated the spinopelvic parameters in patients with FFPs. The baseline characteristics are typical for patients with FFPs. Our hypothesis, that the spinopelvic parameters of the present study cohort differ significantly from asymptomatic patients, was confirmed. PI and PT are significantly increased in patients with FFPs. LL, LLI and SS are significantly reduced. The limitations of this study include the retrospective nature of single-center data collection and the lack of an age-matched control population. Due to the retrospective study design, no functional outcome parameters were analyzed. The results of the present study underline the importance of the spinopelvic alignment and pelvic morphology in the development of FFPs. More studies are needed to further investigate the exact contribution of these parameters in patients with FFPs.

## 5. Conclusions

The spinopelvic parameters in patients with FFPs differ significantly from asymptomatic patients. In patients with FFPs, LL, LLI and SS are significantly reduced, and PI and PT are significantly increased. The sagittal spinopelvic balance is abnormal in patients with FFPs.

## Figures and Tables

**Table 1:** The baseline characteristics of the study population are typical for patients with an FFP.

Patients	Mean Age (Years)	IQR (Years)
Study population	81.0	77.1–84.8
Female patients	80.9	76.9–85.3
Male patients	81.5	79.1–84.9

**Table 2:** This tables presents the mean parameters of LL, PT, SS, PI and LLI.

Parameters	Degree	SD (Degree)
LL	47.9	±14.4
PT	29.4	±8.8
SS	34.2	±10.4
PI	64.4	±12.9
LLI	0.7	±0.2

**Table 3:** This table compares the results of the present study with previously published data [33,34,35].

Parameters	Present Study’s Degree	Schwab [33] Degree of Asymptomatic Patients > 60 Years	LeHuec [34] Degree of Asymptomatic Patient	Barrey-Roussouly [35] Degree of Asymptomatic Patients
LL	47.9 ± 14.4	57 ± 11	55.8 ± 10.2	61 ± 9.7
PT	29.4 ± 8.8	16 ± 9	11.6 ± 7.0	12 ± 6.5
SS	34.2 ± 10.4	36 ± 9	40.2 ± 7.7	40 ± 8.2
PI	64.4 ± 12.9	51 ± 9	52.0 ± 10.5	52 ± 10.7
LLI	0.7 ± 0.2	1.1	1.1	1.2

## Data Availability

Data are contained within the article.

## Appendix A

**Table A1:** The parameter of the study population are listed in this table.

LL [L1-S1]	PT	SS	PI	FFP Classification/ OF-Pelvis Classification
66	19.8	46.15	67.75	FFP II/OF3
32.15	22.65	23.05	47.85	FFP I/OF2
55.85	30.9	41.85	71.6	FFP II/OF3
51.35	34	38.3	74.1	FFP II/OF3
43.15	27.2	23.7	53.15	FFP II/OF3
75.3	45.5	44.05	100.9	FFP II/OF3
22.05	42.35	30.7	72.8	FFP II/OF3
38.2	39.65	25.75	66.65	FFP II/OF3
42.2	20.8	27.9	51.05	FFPII/OF3
42.35	46.35	31.55	75.55	FFP IV/OF5
23.75	38.9	23.9	63.5	FFP II/OF3
36.9	37.15	27.35	63.4	FFP II/OF3
42.25	21.8	35.3	57.25	FFP II/OF3
59.9	31.15	45.15	77.4	FFP II/OF3
21.05	25.75	13.7	43.25	FFP II/OF3
32.55	28.1	27	54.9	FFP II/OF3
54.65	23.8	39.8	64.65	FFP IV/OF5
53	20.1	27.1	50.2	FFP II/OF3
82.9	35.7	64	99.55	FFP II/OF3
36.05	24.65	33.3	58	FFP II/OF3
45.85	28.55	29.55	58.55	FFP II/OF3
49.85	28.15	41.15	68.85	FFP II/OF3
21.95	38.3	13.3	49.7	FFP II/OF3
31.2	23.65	29.95	53.35	FFP II/OF3
45.9	30.15	30	61.4	FFP I/OF2
44.7	37.9	22.9	63.15	FFP II/OF3
33.5	19.6	30.25	50.5	FFP II/OF3
63.35	30.25	47	77	FFP I/OF2
50.75	18.05	39.2	55.5	FFP II/OF3
53.3	25.8	49.75	75.8	FFP II/OF3
64.95	35.25	45	81.8	FFP II/OF3
50.6	20.2	30.65	51.35	FFP II/OF3
68.55	21.2	46.1	67.65	FFP I/OF2
57.5	35.55	34.8	71.65	FFP II/OF3
49.05	36.85	20.5	59.55	FFP I/OF2
54.2	12.1	41.05	53.95	FFP II/OF3
40.5	31.1	25.35	58.05	FFP II/OF3
53	24.2	36.65	60.65	FFP II/OF3
31.95	34.55	34.4	70.4	FFP I/OF2
40.5	35.95	28.15	64.05	FFP IV/OF5
39.9	36.4	25.25	60.9	FFP II/OF3
50.55	22.15	26.55	49.75	FFP II/OF3
66.05	31.05	48.75	83.05	FFP II/OF3
65.9	34.95	52.75	80.85	FFP I/OF2
52.65	11	45.95	57.1	FFP II/OF3
60.2	41.65	42.75	86.7	FFP II/OF3
40.65	52.05	33.5	83.75	FFP II/OF3
47.4	34.85	33.9	68.1	FFPII/OF3
61.25	11.6	38.55	50.6	FFP II/OF3
67.05	21.3	49.85	73.15	FFP IV/OF5
57.95	37.8	41.25	79.35	FFP II/OF3
50.3	24.95	33.65	60.65	FFP II/OF3
6.75	28.65	11	40.35	FFP II/OF3
53.55	27.7	33.65	61.75	FFP II/OF3
49.05	19.45	28.2	51.95	FFP II/OF3
49.65	31.05	27.15	60.95	FFP I/OF2
49.1	26.95	29.45	56.7	FFP III/OF4
